# Dispensing of zolpidem and benzodiazepines in Brazilian private pharmacies: a retrospective cohort study from 2014 to 2021

**DOI:** 10.3389/fphar.2024.1405838

**Published:** 2024-11-11

**Authors:** Fabiana Carvalho, André Comiran Tonon, Maria Paz Hidalgo, Manuela Martins Costa, Sotero Serrate Mengue

**Affiliations:** ^1^ Programa de Pós-Graduação em Epidemiologia, Faculdade de Medicina, Universidade Federal do Rio Grande do Sul, Porto Alegre, Brazil; ^2^ Laboratório de Cronobiologia e Sono, Hospital de Clínicas de Porto Alegre, Universidade Federal do Rio Grande do Sul, Porto Alegre, Brazil

**Keywords:** zolpidem, z-drugs, benzodiazepines, sleep disorders, insomnia, psychotropic drugs, pharmacoepidemiology

## Abstract

**Objective:**

The study aimed to evaluate Zolpidem and Benzodiazepines prescription and dispensing data in private pharmacies in Brazil from 2014 to 2021. Methods: This retrospective cohort study was carried out with retrospective open data from the Brazilian Federal Government from January 2014 to August 2021 containing medicines registered in the National Controlled Products Management System (SNGPC).

**Results:**

Between January 2014 and August 2021, a total of 32,441,392 sales of thirteen drugs from the z-drug and benzodiazepine classes used to treat sleep disorders were recorded in Brazil. Throughout the entire period, clonazepam emerged as the most popular drug, accounting for 29.8% of total sales. Alprazolam followed in second place with 20.6% of sales, while zolpidem came in third with 14.4%. The normal-release form of zolpidem was consistently the highest-selling variant during the evaluation period. However, the fast-acting-release form exhibited the most significant growth, indicated by a noticeable upward trend in sales since 2020. In contrast, the extended-release form of zolpidem remained stable over the years.

**Conclusion:**

The increased sales of zolpidem in Brazilian private pharmacies raise concerns about potential misuse and dependence on this drug mainly for the treatment of insomnia. The epidemic of sleeping pills arises in a scenario of expectancy of short-term amelioration of symptoms, with no correspondence in best clinical practice. Education and counseling for both healthcare professionals and the general population are essential to address this growing health concern and ensure the safe and appropriate use of medications for sleep disorders.

## 1 Introduction

Insomnia is a common and chronic sleep–wake disorder that has a significant impact on patients and healthcare systems. In Brazil, sleep disorders account for almost a quarter of health problems and can impact education, income, and work (Etindele Sosso et al., 2023).

Surveys of the Brazilian population indicate that up to 76% of the studied populations suffer from at least one sleep complaint, indicating that approximately 108 million Brazilians may be affected by sleep disorders (Bittencourt et al., 2009; Hirotsu et al., 2014). Specifically, Insomnia would be represented by up to 45% of such population, which indicates around 64 million people in Brazil (Bittencourt et al., 2009; Castro et al., 2013; Hirotsu et al., 2014). Analyzing over 60 thousand individuals in a nation-representative sample, Kodaira and Silva (Kodaira and Silva, 2017) describe a prevalence of sleeping pill use of 7.6%, with an average treatment duration of almost 10 years and a description of self-medication in 11.2% of the users.

There are both non-pharmacological and pharmacological interventions available to treat insomnia. However, medications are often preferred due to their accessibility and quick response time, even though they have a greater risk of adverse events compared to non-pharmacological interventions like sleep hygiene and cognitive behavioral therapy (De Crescenzo et al., 2022).

Two classes of medications commonly used to treat insomnia are benzodiazepines and z-drugs. Benzodiazepines function as allosteric agonists of Gamma-Amino Butyric Acid-A (GABA-A) receptors, enhancing neurotransmitter effects and leading to neuronal hyperpolarization. Their effects depend on the distribution of receptor subunits within the central nervous system, producing anxiolytic, hypnotic, muscle-relaxant, amnesic, antiepileptic, and respiratory depressant outcomes. In the mesolimbic system, inhibition of GABA increases dopaminergic signaling, contributing to the reward effect associated with abuse and dependence (Drager et al., 2023).

Benzodiazepines can be effective for treating insomnia but pose significant risks, such as tolerance, dependence, and abuse, which makes them unsuitable as a first-line option. The choice of benzodiazepine should be based on the primary condition, with a careful evaluation of the risks versus benefits, prioritizing the lowest effective dose for the shortest possible duration, along with a gradual tapering plan and regular follow-up (De Crescenzo et al., 2022; Drager et al., 2023). A 2012 study of Brazilian data found that the lifetime prevalence of benzodiazepine use was (9.8%), which is high compared to other countries, indicating a greater reliance on benzodiazepines in Brazil for managing conditions such as anxiety and insomnia (Madruga et al., 2018).

Although structurally distinct from benzodiazepines, z-drugs produce their hypnotic effects by binding to benzodiazepine receptors, thereby enhancing the activity of the inhibitory neurotransmitter GABA (De Crescenzo et al., 2022). The best-known representative of z-drugs is zolpidem, which was introduced onto the market in the 1990s and approved only for the treatment of insomnia (Brandt and Leong, 2017). Zolpidem is a short-acting hypnotic drug that enhances the activity of the inhibitory GABA-A receptors, thus inducing sleep (De Crescenzo et al., 2022). Zolpidem effectively decreases sleep onset time and prolongs sleep duration while also being associated with minimal daytime drowsiness, rendering it a suitable option for occasional and short-term insomnia lasting less than 4 weeks (Brandt and Leong, 2017).

Concerning adverse effects, zolpidem has been linked to a wide variety of uncomfortable and dangerous scenarios. A review of adverse events from z-drugs and benzodiazepines points to serious negative health outcomes, such as sleepwalking and engaging in other complex behaviors during sleep, such as driving, eating, or performing household tasks without subsequent remembrance (Schifano et al., 2019; Food and Administration, 2021; Orsolini et al., 2021; Chiappini et al., 2022). The use of zolpidem has been associated with an increased risk of falls, fractures, and motor vehicle accidents, especially in elderly patients, as a consequence of psychomotor impairment (Park et al., 2015; Brandt and Leong, 2017). Although effective in providing rapid response to sleep disorders, zolpidem has safety and tolerance issues, with a concern about the potential for dependence, and misuse, particularly among individuals who use the medication for extended periods or in high doses (De Crescenzo et al., 2022). Furthermore, discontinuation of zolpidem may lead to a temporary exacerbation of insomnia, referred to as rebound insomnia (Brandt and Leong, 2017).

Despite these short and long-term adverse events leading to patient dropouts (De Crescenzo et al., 2022), zolpidem’s usage worldwide continues to rise significantly (de Lima et al., 2023). The possible misuse and abuse of zolpidem is a serious concern due to the potential health consequences and associated harms, which include behavioral and social problems. Furthermore, recent evidence–mainly case reports and case series–draw attention to its significant dependence and abuse potential (Victorri-Vigneau et al., 2014).

Zolpidem was approved by the Food and Drug Administration (FDA) in 1992, and since then, the FDA released two Drug Safety Communications on zolpidem products (Communication, 2014). These communications described the risk of next-day impairment and recommended lower initial doses, especially for women. After the FDA’s action, several studies reported a reduction in the prescribed dose, which led to a decrease in the risks of adverse effects (Norman et al., 2017). In Brazil, zolpidem has been available since the mid-1990s, but since 2007, when the medication patent expired, its advertising increased. In 2023, there are at least 14 different companies that produce and sell all versions of zolpidem in the Brazilian drug market (Barros et al., 2018). An essential problem regarding the use of zolpidem in Brazil is related to surveillance mechanisms. Zolpidem is among the top-listed falsified prescriptions, and to limit this phenomenon, health authorities and agencies have started to provide different measures and regulations (Jouanjus et al., 2018). In other countries, like France, for example, since April 2017, zolpidem prescriptions have had to be performed on a secured prescription pad, and their prescriptions are limited to 4 weeks, which includes a tapering period (Laforgue et al., 2022).

Following the review conducted so far, we have realized that there is a gap in the literature regarding the detailed trends and patterns of zolpidem sales in Brazil, particularly in comparison to other hypnotics like benzodiazepines. Although some studies have reported increased psychotropic use over time and during the COVID-19 pandemic (Del Fiol et al., 2023; Saavedra et al., 2022), none have specifically analyzed long-term trends in zolpidem sales, including its various formulations, or compared these trends with benzodiazepines over an extended period. Therefore, it is fundamental to understand the sale pattern of this medication. In this way, our research addresses this gap by evaluating Zolpidem and Benzodiazepines prescription and dispensing data in private pharmacies in Brazil from 2014 to 2021.

## 2 Methods

### 2.1 Study design and database

This retrospective cohort study was conducted using retrospective data from the Brazilian Federal Government’s open data website (dados.gov.br), extracted in CSV format on 02/04/2023. All monthly files from January 2014 to August 2021 were included for analysis. The study follows the STrengthening the Reporting of OBservational studies in Epidemiology (STROBE) guidelines to reporting of observational data, with the checklist provided in the Supplementary Material S1 (Von Elm et al., 2007).

The database refers to individual sales made in private pharmacies in Brazil of medicines subject to special control, mainly psychotropic drugs and antibiotics, with sales registered in the National System for Management of Controlled Products (SNGPC) of the Brazilian Health Regulatory Agency (ANVISA). The SNGPC is an electronic platform developed by ANVISA to monitor the sale and inventory of medications and substances subject to special control, such as narcotics, psychotropics, and anabolic steroids. This platform periodically receives data from pharmacies via the web, validates the records, and compiles them into a centralized system. Pharmacies and drugstores in Brazil are required to register all entries and exits of these controlled substances, allowing ANVISA to track their movement through the supply chain. The system captures essential data, including product identification (commercial and chemical names, dosage, and batch numbers), transaction details (purchases, sales, returns), and information on suppliers. This enables continuous monitoring by ANVISA, ensuring regulatory compliance and overseeing the flow of these substances (Brazilian Ministry of Health, 2006). According to ANVISA (Brazilian Health Regulatory Agency, 2018), there are 59,162 pharmacies in Brazil registered with the SNGPC that sell controlled medications.

### 2.2 Database analysis

From this analysis, we extracted variables related to the commercialization records (month/year and state/municipality of the sale), the prescribed medication itself (active ingredient, pharmaceutical form, concentration per dosage unit in mg), and the prescriber (professional council, state of the professional council, and type of prescription). Incomplete or inconsistent records were excluded during the data cleansing process.

The inclusion criteria included medications sold in Brazil from the z-drug classes and benzodiazepines used to treat sleep disorders from private pharmacies in Brazil between January 2014 and August 2021, as recorded in the SNGPC. From the database records, 13 drugs were extracted: Alprazolam, Bromazepam, Clobazam, Clonazepam, Cloxazolam, Diazepam, Estazolam, Flunitrazepam, Flurazepam, Lorazepam, Midazolam, Zolpidem, Zopiclone. In addition, the corresponding pharmaceutical form was grouped into normal release forms (without specification), rapid release (Immediate/Fast-acting), and slow release (Controlled/Extended/Modified). Public pharmacies were not part of the dataset, as their sales were not captured in the system.

The Research Ethics Committee in Brazil did not require approval as the study was conducted using open databases.

### 2.3 Statistical analysis

The data was processed and analyzed using the R package version 4.3.1. In addition to the basic data processing and analysis libraries, the Strucchange, Changepoint, and Segmented packages were used for interrupted time series.

## 3 Results

Between January 2014 and August 2021, private pharmacies in Brazil recorded a total of 32,441,392 sales for thirteen drugs used to treat sleep disorders, including benzodiazepines and z-drugs. Throughout the entire period, clonazepam was the most widely sold drug, accounting for 29.8% of all sales. Alprazolam ranked second with 20.6% followed by zolpidem in third with 14.4% (Table 1).

**TABLE 1:** Number of total sales of the selected drugs.

Medications	Year sale								
	2014	2015	2016	2017	2018	2019	2020	2021	Total
z-Drug									
Zolpidem	338,367	372,606	426,846	473,720	582,034	756,968	910,382	801,353	4,662,276
	(7%)	(8%)	(9%)	(10%)	(13%)	(16%)	(20%)	(17%)	(14.4%)
Zoplicone	15,060	14,987	12,848	13,034	12,067	48,757	91,668	83,910	292,331
	(5%)	(5%)	(4%)	(5%)	(4%)	(17%)	(31%)	(29%)	(0.9%)
Benzodiazepines									
Clonazepam	1,192,211	1,118,066	1,148,740	1,220,230	1,269,476	1,307,195	1,347,121	1,056,520	9,659,559
	(12%)	(12%)	(12%)	(13%)	(13%)	(13%)	(14%)	(11%)	(29.8%)
Alprazolam	787,968	777,804	806,609	851,067	872,068	895,366	938,928	747,395	6,677,205
	(12%)	(12%)	(12%)	(13%)	(13%)	(13%)	(14%)	(11%)	(20.6%)
Bromazepam	523,318	482,101	477,052	480,798	469,313	456,275	448,117	330,466	3,667,440
	(14%)	(13%)	(13%)	(13%)	(13%)	(13%)	(12%)	(9%)	(11.3%)
Diazepam	294,761	276,190	263,079	276,697	265,313	254,185	271,426	202,721	2,104,372
	(14%)	(13%)	(12%)	(13%)	(13%)	(12%)	(13%)	(10%)	(6.5%)
Lorazepam	232,413	209,770	221,555	227,011	224,225	187,793	178,079	130,471	1,611,317
	(14%)	(13%)	(14%)	(14%)	(14%)	(12%)	(11%)	(8%)	(5%)
Clobazam	139,555	127,012	135,210	143,179	149,465	154,350	150,584	118,192	1,117,547
	(12%)	(11%)	(12%)	(13%)	(13%)	(14%)	(13%)	(11%)	(3.4%)
Cloxazolam	282,100	256,607	222,695	198,132	52,811	812	244	121	1,013,522
	(28%)	(25%)	(22%)	(20%)	(5%)	(*<*0.1%)	(*<*0.1%)	(*<*0.1%)	(3.1%)
Flunitrazepam	99,536	95,113	95,768	93,414	90,130	94,626	90,628	66,460	725,675
	(14%)	(13%)	(13%)	(13%)	(12%)	(13%)	(12%)	(10%)	(2.2%)
Midazolam	65,174	62,409	59,372	57,022	54,006	52,152	50,999	37,343	438,477
	(15%)	(14%)	(14%)	(13%)	(12%)	(12%)	(12%)	(8%)	(1.4%)
Flurazepam	42,961	41,599	42,030	42,154	42,188	41,242	41,312	30,908	324,394
	(13%)	(13%)	(13%)	(13%)	(13%)	(13%)	(13%)	(9%)	(1%)
Estazolam	18,018	20,928	22,934	24,756	26,112	19,331	6,303	8,895	147,277
	(12%)	(14%)	(16%)	(17%)	(18%)	(13%)	(4%)	(6%)	(0.4%)
Total	3,369,038 (12%)	3,236,537 (11%)	3,343,881 (12%)	3,529,523 (12%)	3,682,429 (13%)	3,857,782 (14%)	4,094,053 (14%)	3,268,926 (12%)	32,441,392 (100%)

**FIGURE 1:**
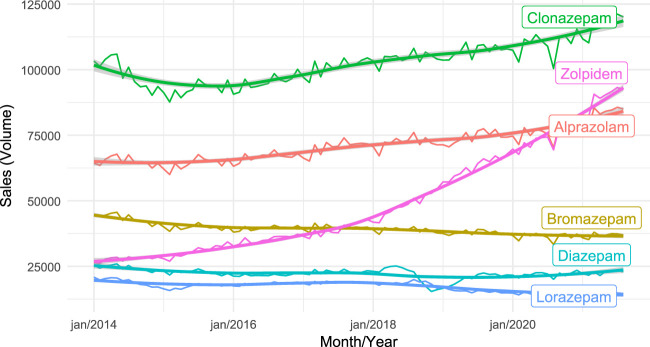
The trend of drug sales over the selected period. The sales of the six drugs are presented separately. The vertical axis displays the sales volume of each drug, and the horizontal axis shows time in months/years.

Figure 1 illustrates the sales of the six drugs with the highest sales volumes. We find that sales of Clonazepam and Alprazolam remained relatively stable, with slight growth towards the end of the period. However, the sales of Diazepam, Bromazepam, and Lorazepam decreased over the years. Zolpidem showed a more significant increase in sales than other drugs, especially in 2018 and 2020. In mid-2020, a slight decline in sales, forming a noticeable valley in Figure 1, was observed across all drugs.

**FIGURE 2:**
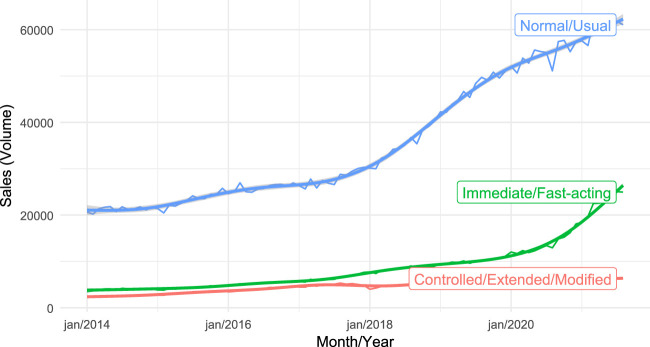
Zolpidem’s sales trend, separated by the three types of presentation: normal-release (blue curve), fast-acting-release (green curve), and extended-release (red curve).

Regarding zolpidem’s sales trend, we present the total sales separated by the three types of presentation: normal-release tablet, fast-acting-release sublingual tablet, and extended-release tablet (Figure 2). The normal-release form is the most sold throughout the period evaluated, but the fast-acting-release form is the one that has been growing the most, with an increase in the inflection of the curve since 2020. The extended form has remained stable over time.

## 4 Discussion

The growth in the use of zolpidem has increased drastically and has shown two peaks with more significant growth inflection, one in 2018 and the second in 2020. Initially, our research analyzed only the sale of zolpidem without a comparison with other psychotropic drugs. However, aiming to verify if the brutal growth of zolpidem occurred due to a substitution of other drugs, we conducted a comparative analysis of the dispensing of medications also used for sleep disorders, particularly insomnia: the Benzodiazepines. After the comparison, we found that the sales pattern of the other drugs analyzed has maintained a constant pattern. Still, zolpidem grows more than the others throughout the analyzed period.

Our findings highlight that sales of zolpidem have been steadily increasing over the 7 years; however, since the COVID-19 pandemic began, the rapid dispensing form of the drug has seen a higher rate of growth, leading to a new pattern in this medication’s consumption, which should be caught with attention. Given zolpidem’s unique pharmacokinetic profile - marked by a much shorter duration of action compared to most commonly used benzodiazepines - the potential for misuse and abuse remains a concern. Preclinical studies have demonstrated that zolpidem exhibits reinforcing effects similar to midazolam, a short-acting benzodiazepine, in nonhuman primates (Huskinson et al., 2019). This suggests that, despite its shorter duration, zolpidem shares abuse-related properties with benzodiazepines, highlighting the need for careful monitoring of its use in clinical settings. In this sense, zolpidem is already a fast-acting drug, and now we have a dispensing form that acts even faster, which takes us dealing with a pattern of use that could be even more potentially addictive.

Our study has identified two growth points in zolpidem sales: in 2018 and 2020. The first inflection point is attributed to the European Sleep Research Society’s publication in 2017 on insomnia treatment (Riemann et al., 2017). This publication is believed to have contributed to the 23% increase in sales in 2018 compared to the previous year. The second inflection point observed was responsible for a 20% increase in zolpidem sales in 2020 compared to 2019. We attribute this increase to the COVID-19 pandemic’s influence on the mental health of the general population, which led to an increased consumption of psychotropic drugs. This trend aligns with Saavedra’s study (Saavedra et al., 2022). Furthermore, in mid-2020, a slight decline in sales was observed, as shown in Figure 1, for the six drugs with the highest sales volumes. This is likely due to the social isolation measures imposed during the pandemic, which impacted the frequency of elective consultations, as well as possible delays in the registration of sales in the SNGPC system caused by the pandemic.

It is important to note that the study period (2014–2021) may no longer fully reflect the current situation. As mentioned earlier, the global COVID-19 pandemic, particularly the lockdowns and isolation measures, significantly impacted sleep and mental health worldwide (Sousa et al., 2021) and in Brazil (Schäfer et al., 2022). The long-term effects of this unprecedented event on sleep patterns are still not fully understood. However, it is reasonable to assume that the widespread use of zolpidem during the pandemic has continued into the post-pandemic period, especially given the concerning rates of addiction to this drug reported globally following the emergence of COVID-19 (de Lima et al., 2024).

Moreover, specific recommendations for certain populations, such as starting with lower starting doses of zolpidem, particularly for women, are based on the risk of daily impairment. These risks, which include car accidents, falls, and fractures (Brandt and Leong, 2017), are similar to those associated with benzodiazepines. However, some individuals might be using higher dosages due to tolerance (Barros et al., 2018), raising concerns about the steep increase in the use of this medication. While zolpidem is effective in shortening sleep latency and prolonging sleep duration, it presents significant safety and tolerance concerns, particularly with long-term use. These concerns are compounded by its effects on sleep architecture: zolpidem has been shown to decrease rapid eye movement (REM) sleep and increase slow-wave sleep (SWS), leading to non-physiological sleep patterns over time (Bettica et al., 2012). This disruption of natural sleep cycles raises concerns about the long-term health consequences of chronic zolpidem use, particularly in older adults.

These concerns align with the pharmacological profile of both benzodiazepines and zolpidem. Both drugs exert their effects primarily through the modulation of GABAA receptors, particularly those containing the α 1 subunit, which is crucial for the development of tolerance and dependence. Preclinical studies, such as those by Duke et al. (Duke et al., 2021), have shown that chronic benzodiazepine use leads to rapid tolerance to sedative and ataxic effects mediated by α 1GABAA receptors. Furthermore, these studies suggest that physical dependence is closely linked to α 1GABAA receptors, as significant withdrawal symptoms occur when α 1-specific antagonists are administered. Given zolpidem’s selective affinity for α 1GABAA receptors, it likely induces similar adverse effects, including tolerance and dependence, mirroring the outcomes observed with chronic benzodiazepine use. This highlights the need for caution when prescribing zolpidem for long-term use, due to its potential to elicit effects akin to those seen with benzodiazepines.

Using the GRADE approach (Grades of Recommendation, Assessment, Development and Evaluation) (Morgenthaler et al., 2016), the American Academy of Sleep Medicine evaluated the level of recommendation of hypnotics compared to placebo as treatment for sleep onset or sleep maintenance insomnia. Their most recent guideline shows that the recommendations for all pharmacologic agents (including zolpidem) are weak, meaning a lower degree of clinical certainty (Sateia et al., 2017). Indeed, cognitive behavioral therapy for insomnia (CBT-i) is still the only intervention strongly recommended as first-line treatment for chronic insomnia in adults of any age, with evidence of improvements that are sustained over time (Morin et al., 2020; Riemann et al., 2017). The overall consensus is that CBT-i is the only therapeutic approach with high-quality evidence and that a pharmacological intervention should be offered if CBT-i is not sufficiently effective or not available.

Considering that the observed increase in the purchase of zolpidem is necessarily attached to an increase in the prescription of this medication, it is possible to assume that doctors either ignore or are unfamiliar with up-to-date evidence. An additional hypothesis that can be drawn upon the possibility of unavailability of CBT-i. This assertion would be true if buyers represent users of the public healthcare system, since psychotherapeutic approaches are usually not accessible to populations of low and middle-income countries that rely on public healthcare providers (Beck et al., 2016; Verhey et al., 2020). However, the present data is based on the purchase of zolpidem in private pharmacies, once they are not subsidized by the public system – as benzodiazepines are. Hence, our data might represent a significant portion of users from the private sector, where psychotherapy is widely available, but often overlooked and underused (Blay et al., 2014).

According to the guidelines of the European Sleep Research Society, zolpidem should be prescribed for short-term treatment of insomnia (i.e., ≤4 weeks) (Riemann et al., 2017). One could argue that the prescription of sleeping pills analyzed in this study would be more representative of the population suffering from acute insomnia. Nevertheless, several cohorts report that insomnia symptoms are present in up to 74% of the cases in a 1-year follow-up and nearly 40% in three or 5 years of follow-up (Morin et al., 2021; 2020; Morphy et al., 2007).

As mentioned, the use of zolpidem for a duration longer than 4 weeks might be a result of the continuous prescription of healthcare professionals because of unfamiliarity with evidence or unavailability of CBT-i. However, the chronic use of zolpidem might be a result of dependence or misuse. Growing evidence indicates the pharmacological potential for and the clinical syndrome of dependence to zolpidem (Barros et al., 2018; Victorri-Vigneau et al., 2014). On the other side, it is known that hypnotics misuse (and not dependence) might result from unfavorable attitude toward sleep (Yen et al., 2015a; b), lack of knowledge regarding good sleep practices and sleep hygiene, or cognitive distortions (Furukawa et al., 2024) – for example, most individuals with acute insomnia will show irregular sleep scheduling and the fear of not sleeping. These factors are significant contributors to the perpetuation and chronification of insomnia syndrome, and they all represent treatment targets of CBT-i but not pharmacological agents (Morin and Benca, 2012).

This study’s findings had some limitations: first, sales records do not necessarily indicate the population’s actual consumption of these medicines. Therefore, we consider the dispensing as an estimate of participants’ consumption data. Second, our data does not allow us to determine the quantity of zolpidem sold per individual. As a result, it is not possible for us to estimate the total usage duration of an individual or infer dependence based on this population. Third, only sales from private pharmacies were counted, and public consumption was not recorded. This limitation prevented us from getting a complete view of the general Brazilian panorama of the growth in the sale of these drugs, which may have led to underestimating our numbers. Finally, we do not have information about the specific medical condition for which the medications were prescribed. This limitation may overestimate the issue of insomnia, as some drugs may have been prescribed for another clinical condition, such as anxiety, for example.

## 5 Conclusion

Zolpidem is suitable for the short-term treatment of insomnia. However, our research has found a considerable rise in the sales of this drug in Brazil’s private pharmacies over the 7 years analyzed. This increase may suggest the medication is prescribed longer than the recommended 4 weeks for acute insomnia. In this case, the first-line treatment would be CBTi and not the use of sedatives, which suggests a pattern of prescription that goes against current evidence. This prescriptive behavior may be a result of the physician’s lack of familiarity with the evidence or the local unavailability of CBT-i.

Finally, this study portrays a dispensing profile of the rapid-release form that highlights a potential and dangerous issue regarding the use of zolpidem: dependence or misuse. The epidemic of sleeping pills arises in a scenario of expectancy of short-term amelioration of symptoms, with no correspondence in best clinical practice. Therefore, this is a growing health concern that requires education and counseling of the population as much as adequate training and attention of healthcare professionals.

## Data Availability

Publicly available datasets were analyzed in this study. This data can be found here: https://dados.gov.br/dataset?q&equals;anvisa.

## References

[B1] Barros V. V. d. Opaleye E. S. Noto A. R. (2018). Is the regulation of z-drugs in Brazil in line with scientific research and international standards? Brazilian. J. Psychiatry 40, 112. 10.1590/1516-4446-2017-2372 PMC6899425 29590268

[B2] Beck A. Nadkarni A. Calam R. Naeem F. Husain N. (2016). Increasing access to cognitive behaviour therapy in low and middle income countries: a strategic framework. Asian J. psychiatry 22, 190–195. 10.1016/j.ajp.2015.10.008 26643366

[B3] Bettica P. Squassante L. Groeger J. A. Gennery B. Winsky-Sommerer R. Dijk D.-J. (2012). Differential effects of a dual orexin receptor antagonist (sb-649868) and zolpidem on sleep initiation and consolidation, sws, rem sleep, and eeg power spectra in a model of situational insomnia. Neuropsychopharmacology 37, 1224–1233. 10.1038/npp.2011.310 22237311 PMC3306884

[B4] Bittencourt L. R. A. Santos-Silva R. Taddei J. A. Andersen M. L. de Mello M. T. Tufik S. (2009). Sleep complaints in the adult brazilian population: a national survey based on screening questions. J. Clin. Sleep Med. 5, 459–463. 10.5664/jcsm.27603 19961032 PMC2762719

[B5] Blay S. L. Fillenbaum G. G. da Silva P. F. R. Peluso E. T. (2014). Use of psychotherapy in a representative adult community sample in são paulo, Brazil. J. Nerv. Ment. Dis. 202, 688–694. 10.1097/NMD.0000000000000181 25118139 PMC4149600

[B6] Brandt J. Leong C. (2017). Benzodiazepines and z-drugs: an updated review of major adverse outcomes reported on in epidemiologic research. Drugs R&D 17, 493–507. 10.1007/s40268-017-0207-7 PMC5694420 28865038

[B7] Brazilian Health Regulatory Agency (ANVISA) (2018). Brasil tem 59 mil farmácias que vendem controlados. Available at: https://www.gov.br/anvisa/pt-br/assuntos/noticias-anvisa/2018/brasil-tem-59-mil-farmacias-que-vendem-controlados.

[B8] Castro L. S. Poyares D. Leger D. Bittencourt L. Tufik S. (2013). Objective prevalence of insomnia in the são paulo, Brazil epidemiologic sleep study. Ann. neurology 74, 537–546. 10.1002/ana.23945 23720241

[B9] Chiappini S. Schifano F. Martinotti G. (2022). Editorial: prescribing psychotropics: misuse, abuse, dependence, withdrawal and addiction, Volume II. Front. Psychiatry 13, 1053896. 10.3389/fpsyt.2022.1053896 37255962 PMC10226078

[B10] Communication F. D. S. (2014). Fda approves new label changes and dosing for zolpidem products and a recommendation to avoid driving the day after using ambien cr. Food Drug Adm. Drug Saf. Commun. Available at: https://www.fda.gov/drugs/drugsafety-and-availability/fda-drugsafety-communication-fda-approvesnew-label-changes-and-dosingzolpidem-products-and .

[B11] [Dataset] Brazilian Ministry of Health, B (2006). “implantação do sistema nacional de gerenciamento de produtos controlados sngpc: guia de credenciamento no sngpc,” in Projeto sngpc para farmácias e drogarias.

[B12] De Crescenzo F. D’Alò G. L. Ostinelli E. G. Ciabattini M. Di Franco V. Watanabe N. (2022). Comparative effects of pharmacological interventions for the acute and long-term management of insomnia disorder in adults: a systematic review and network meta-analysis. Lancet 400, 170–184. 10.1016/S0140-6736(22)00878-9 35843245

[B13] Del Fiol F. d. S. Bergamaschi C. d. C. Lopes L. C. Silva M. T. Barberato-Filho S. (2023). Sales trends of psychotropic drugs in the covid-19 pandemic: a national database study in Brazil. Front. Pharmacol. 14, 1131357. 10.3389/fphar.2023.1131357 37007033 PMC10063839

[B14] de Lima W. D. da Silva M. D. de Souza Costa E. Pinheiro F. I. de Azevedo E. P. Cobucci R. N. (2023). Abusive use of zolpidem as a result of covid-19 and perspectives of continuity of the problem in the post-pandemic period. Curr. Neuropharmacol. 22, 1578–1582. 10.2174/1570159X21666230920123401 PMC11284723 37811654

[B15] de Lima W. D. da Silva M. D. de Souza Costa E. Pinheiro F. I. de Azevedo E. P. Cobucci R. N. (2024). Abusive use of zolpidem as a result of covid-19 and perspectives of continuity of the problem in the post-pandemic period. Curr. Neuropharmacol. 22, 1578–1582. 10.2174/1570159X21666230920123401 37811654 PMC11284723

[B16] Drager L. F. Assis M. Bacelar A. F. R. Poyares D. L. R. Conway S. G. Pires G. N. (2023). 2023 guidelines on the diagnosis and treatment of insomnia in adults–brazilian sleep association. Sleep Sci. 16, 507–549. 10.1055/s-0043-1776281 38370879 PMC10869237

[B17] Duke A. N. Tiruveedhula V. P. B. Sharmin D. Knutson D. E. Cook J. M. Platt D. M. (2021). Tolerance and dependence following chronic alprazolam treatment in rhesus monkeys: role of gabaa receptor subtypes. Drug alcohol dependence 228, 108985. 10.1016/j.drugalcdep.2021.108985 34500240 PMC8595788

[B18] Etindele Sosso F. Torres Silva F. Queiroz Rodrigues R. Carvalho M. M. Zoukal S. Zarate G. C. (2023). Prevalence of sleep disturbances in Latin american populations and its association with their socioeconomic status—a systematic review and a meta-analysis. J. Clin. Med. 12, 7508. 10.3390/jcm12247508 38137577 PMC10743597

[B19] Food U. Administration D. (2021). Fda requires stronger warnings about rare but serious incidents related to certain prescription insomnia medicines. Available at: https://www.fda.gov/newsevents/press-announcements/fdarequires-stronger-warnings-aboutrare-serious-incidents-related-certainprescription-insomnia .

[B20] Furukawa Y. Sakata M. Yamamoto R. Nakajima S. Kikuchi S. Inoue M. (2024). Components and delivery formats of cognitive behavioral therapy for chronic insomnia in adults: a systematic review and component network meta-analysis. JAMA psychiatry 81, 357–365. 10.1001/jamapsychiatry.2023.5060 38231522 PMC10794978

[B45] Huskinson S. L. Freeman K. B. Rowlett J. K. (2019). Self-administration of benzodiazepine and cocaine combinations by male and female rhesus monkeys in a choice procedure: Role of α1 subunit–containing GABA a receptors. Psychopharmacology 236, 3271–3279. 31183518 10.1007/s00213-019-05286-0 PMC6832789

[B21] Hirotsu C. Bittencourt L. Garbuio S. Andersen M. L. Tufik S. (2014). Sleep complaints in the brazilian population: impact of socioeconomic factors. Sleep Sci. 7, 135–142. 10.1016/j.slsci.2014.08.001 26483918 PMC4559590

[B22] Jouanjus E. Guernec G. Lapeyre-Mestre M. Network F. A. Daveluy A. Le Boisselier R. (2018). Medical prescriptions falsified by the patients: a 12-year national monitoring to assess prescription drug diversion. Fundam. and Clin. Pharmacol. 32, 306–322. 10.1111/fcp.12356 29436015

[B23] Kodaira K. Silva M. T. (2017). Sleeping pill use in Brazil: a population-based, cross-sectional study. BMJ open 7, e016233. 10.1136/bmjopen-2017-016233 PMC5541607 28698341

[B24] Laforgue E.-J. Istvan M. Schreck B. Mainguy M. Jolliet P. Grall-Bronnec M. (2022). Perception of the regulatory change for zolpidem prescription by French general practitioners and its relation to prescription behavior. J. Clin. Med. 11, 2176. 10.3390/jcm11082176 35456269 PMC9032177

[B25] Madruga C. S. Paim T. L. Palhares H. N. Miguel A. C. Massaro L. T. Caetano R. (2018). Prevalence of and pathways to benzodiazepine use in Brazil: the role of depression, sleep, and sedentary lifestyle. Braz. J. Psychiatry 41, 44–50. 10.1590/1516-4446-2018-0088 30328968 PMC6781701

[B26] Morgenthaler T. I. Deriy L. Heald J. L. Thomas S. M. (2016). The evolution of the aasm clinical practice guidelines: another step forward. J. Clin. sleep Med. 12, 129–135. 10.5664/jcsm.5412 26518707 PMC4702195

[B27] Morin C. M. Benca R. (2012). Chronic insomnia. Lancet 379, 1129–1141. 10.1016/S0140-6736(11)60750-2 22265700

[B28] Morin C. M. Inoue Y. Kushida C. Poyares D. Winkelman J. Members G. C. (2021). Endorsement of european guideline for the diagnosis and treatment of insomnia by the world sleep society. Sleep. Med. 81, 124–126. 10.1016/j.sleep.2021.01.023 33667998

[B29] Morin C. M. Jarrin D. C. Ivers H. Mérette C. LeBlanc M. Savard J. (2020). Incidence, persistence, and remission rates of insomnia over 5 years. JAMA Netw. open 3, e2018782. 10.1001/jamanetworkopen.2020.18782 33156345 PMC7648256

[B30] Morphy H. Dunn K. M. Lewis M. Boardman H. F. Croft P. R. (2007). Epidemiology of insomnia: a longitudinal study in a UK population. Sleep 30, 274–280. 10.1093/sleep/30.3.274 17425223

[B31] Norman J. L. Fixen D. R. Saseen J. J. Saba L. M. Linnebur S. A. (2017). Zolpidem prescribing practices before and after food and drug administration required product labeling changes. SAGE Open Med. 5, 2050312117707687. 10.1177/2050312117707687 28515934 PMC5423710

[B32] Orsolini L. Chiappini S. Grandinetti P. Bruschi A. Testa R. Provenzano A. (2021). ‘z-trip’? a comprehensive overview and a case-series of zolpidem misuse. Clin. Psychopharmacol. Neurosci. 19, 367–387. 10.9758/cpn.2021.19.2.367 33888666 PMC8077048

[B33] Park H. Satoh H. Miki A. Urushihara H. Sawada Y. (2015). Medications associated with falls in older people: systematic review of publications from a recent 5-year period. Eur. J. Clin. Pharmacol. 71, 1429–1440. 10.1007/s00228-015-1955-3 26407688

[B34] Riemann D. Baglioni C. Bassetti C. Bjorvatn B. Dolenc Groselj L. Ellis J. G. (2017). European guideline for the diagnosis and treatment of insomnia. J. sleep Res. 26, 675–700. 10.1111/jsr.12594 28875581

[B35] Saavedra P. A. E. Galato D. de Souza Silva C. M. da Silva I. C. R. da Silva E. V. (2022). Dispensing of psychotropic drugs in the brazilian capital city before and during the covid-19 pandemic (2018–2020). Front. Pharmacol. 13, 1028233. 10.3389/fphar.2022.1028233 36618914 PMC9822257

[B36] Sateia M. J. Buysse D. J. Krystal A. D. Neubauer D. N. Heald J. L. (2017). Clinical practice guideline for the pharmacologic treatment of chronic insomnia in adults: an american academy of sleep medicine clinical practice guideline. J. Clin. sleep Med. 13, 307–349. 10.5664/jcsm.6470 27998379 PMC5263087

[B37] Schäfer A. A. Santos L. P. Manosso L. M. Quadra M. R. Meller F. O. (2022). Relationship between sleep duration and quality and mental health before and during covid-19 pandemic: results of population-based studies in Brazil. J. Psychosomatic Res. 158, 110910. 10.1016/j.jpsychores.2022.110910 PMC8993422 35427941

[B38] Schifano F. Chiappini S. Corkery J. M. Guirguis A. (2019). An insight into z-drug abuse and dependence: an examination of reports to the european medicines agency database of suspected adverse drug reactions. Int. J. Neuropsychopharmacol. 22, 270–277. 10.1093/ijnp/pyz007 30722037 PMC6441128

[B39] Sousa G. M. d. Tavares V. D. d. O. de Meiroz Grilo M. L. P. Coelho M. L. G. Lima-Araújo G. L. d. Schuch F. B. (2021). Mental health in covid-19 pandemic: a meta-review of prevalence meta-analyses. Front. Psychol. 12, 703838. 10.3389/fpsyg.2021.703838 34621212 PMC8490780

[B40] Verhey I. J. Ryan G. K. Scherer N. Magidson J. F. (2020). Implementation outcomes of cognitive behavioural therapy delivered by non-specialists for common mental disorders and substance-use disorders in low-and middle-income countries: a systematic review. Int. J. Ment. Health Syst. 14, 40. 10.1186/s13033-020-00372-9 32514304 PMC7260765

[B41] Victorri-Vigneau C. Gérardin M. Rousselet M. Guerlais M. Grall-Bronnec M. Jolliet P. (2014). An update on zolpidem abuse and dependence. J. Addict. Dis. 33, 15–23. 10.1080/10550887.2014.882725 24467433

[B42] Von Elm E. Altman D. G. Egger M. Pocock S. J. Gøtzsche P. C. Vandenbroucke J. P. (2007). The strengthening the reporting of observational studies in epidemiology (strobe) statement: guidelines for reporting observational studies. lancet 370, 1453–1457. 10.1016/S0140-6736(07)61602-X 18064739

[B43] Yen C.-F. Ko C.-H. Chang Y.-P. Yu C.-Y. Huang M.-F. Yeh Y.-C. (2015a). Dependence, misuse, and beliefs regarding use of hypnotics by elderly psychiatric patients taking zolpidem, estazolam, or flunitrazepam. Asia-Pacific Psychiatry 7, 298–305. 10.1111/appy.12147 25296384

[B44] Yen C.-F. Yen C.-N. Ko C.-H. Hwang T.-J. Chen C.-S. Chen T.-T. (2015b). Correlates of dependence and beliefs about the use of hypnotics among zolpidem and zopiclone users. Subst. use and misuse 50, 350–357. 10.3109/10826084.2014.980955 25458710

